# *GSTT1* and *GSTM1* null variants in Mestizo and Amerindian populations from northwestern Mexico and a literature review

**DOI:** 10.1590/1678-4685-GMB-2016-0142

**Published:** 2017-11-06

**Authors:** Luz Elena Palma-Cano, Emilio J. Córdova, Lorena Orozco, Angélica Martínez-Hernández, Miguel Cid, Irene Leal-Berumen, Angel Licón-Trillo, Ruth Lechuga-Valles, Mauricio González-Ponce, Everardo González-Rodríguez, Verónica Moreno-Brito

**Affiliations:** 1 Department of Biochemistry, Faculty of Medicine and Biomedical Science, Autonomus University of Chihuahua, Chihuahua, Chihuahua, Mexico; 2 Department of Clinical Research, National Institute of Genomic Medicine, Mexico City, Mexico; 3 Department of Molecular Biology, Faculty of Zootechnics and Ecology, Autonomus University of Chihuahua, Chihuahua, Chihuahua, Mexico

**Keywords:** Oxidative stress, GSTT1, GSTM1, null variants

## Abstract

The *GSTT1* and *GSTM1* genes are key molecules in cellular detoxification. Null variants in these genes are associated with increase susceptibility to developing different types of cancers. The aim of this study was to determine the prevalence of *GSTT1* and *GSTM1* null genotypes in Mestizo and Amerindian individuals from the Northwestern region of Mexico, and to compare them with those reported worldwide. *GSTT1* and *GSTM1* null variants were genotyped by multiplex PCR in 211 Mestizos and 211 Amerindian individuals. Studies reporting on frequency of *GSTT1* and *GSTM1* null variants worldwide were identified by a PubMed search and their geographic distribution were analyzed. We found no significant differences in the frequency of the null genotype for *GSTT1* and *GSM1* genes between Mestizo and Amerindian individuals. Worldwide frequencies of the *GSTT1* and *GSTM1* null genotypes ranges from 0.10 to 0.51, and from 0.11 to 0.67, respectively. Interestingly, in most countries the frequency of the *GSTT1* null genotype is common or frequent (76%), whereas the frequency of the *GSMT1* null genotype is very frequent or extremely frequent (86%). Thus, ethnic-dependent differences in the prevalence of *GSTT1* and *GSTM1* null variants may influence the effect of environmental carcinogens in cancer risk.

## Introduction

The family of the glutathione S-transferases (GSTs) is composed of enzymes that play an essential role in the cellular protection against a wide range of hazardous molecules, such as reactive oxygen species (ROS), xenobiotics and electrophilic compounds. The mammalian GSTs can be classified into three groups: cytosolic, mitochondrial and membrane-associated proteins in eicosanoid and glutathione metabolism (MAPEG). Cytoplasmic enzymes are further subdivided into seven groups: Alpha (GSTA), Mu (GSTM), Omega (GSTO), Pi (GSTP), Sigma (GSTS), Theta (GSTT), and Zeta (GSTZ) (Tew and Townsend, 2012). Since the individual GSTs proteins can share ligands, functional redundancy is a common event in the GST-mediated biotransformation of toxic compounds (Luo *et al.*, 2011).

GSTs catalyze the conjugation of reduced glutathione (GSH), the major antioxidant molecule in the cell, to a myriad of hazardous molecules, including carcinogens, drugs and xenobiotics. GSH-conjugated substrates are then transported out of the cell mainly via the ABC (ATP-binding cassette) efflux pumps. Additionally, GSTs are able to detoxify noxious products of the cellular metabolism, such as reactive oxygen and nitrogen species through their glutathione peroxidase activity (Board and Menon, 2013; Galal *et al.*, 2015). These enzymes are involved in cellular processes others than detoxification, including chaperone activities, regulation of kinase-mediated signal transduction and S-glutathionylation cycle (Pajaud *et al.*, 2012; Klaus *et al.*, 2013; Zhang *et al.*, 2014a).

Early studies highlight the presence of deletion variants (null variants) in the *GSTM1* and *GSTT1* genes, which are located at chromosomal positions 1p13.3 and 22q11.23, respectively. Individuals with the homozygous genotype for the deletion variants (null/null) in *GSTM1* or *GSTT1* genes showed the total loss of enzymatic activity of the respective protein (Pemble *et al.*, 1994; Xu *et al.*, 1998). In accordance with their detoxification properties, the deficiency of *GSTM1* and *GSTT1*, either individually or in combination, greatly increases the susceptibility of developing cancer in different organs, including liver, lung and colon (Csejtei *et al.*, 2008; Sui *et al.*, 2014; Zhang *et al.*, 2014b).

The prevalence of *GSTM1* and *GSTT1* null alleles shows strong variation among different ethnic groups. For instance, the frequency of the *GSTM1* null allele was as low as 0.23 in South Africa, but up to 0.42 in Spain and 0.67 in Singapore (Masimirembwa *et al.*, 1998; Chan *et al.*, 2011; Ruano-Ravina *et al.*, 2014). With regard to *GSTT1*, the frequency of the null genotype among Greek individuals was 0.10, whereas in England and Japan the frequency was 0.21 and 0.50, respectively (Garte *et al.*, 2001; Dialyna *et al.*, 2003; Hishida *et al.*, 2005). These differences could modulate the risk to different types of tumors in populations of different ethnic ancestry. For instance, Japan, one of the countries with the highest frequency of the null genotype for both *GSTM1* and *GSTT1* genes, has a high incidence of colorectal, stomach, esophagus and prostate cancer (WHO, 2012). Although studies about the distribution of *GSTM1* and *GSTT1* null genotypes in a Mexican-Mestizo population have been performed previously (Pérez-Morales *et al.*, 2008; Pérez-Morales *et al.*, 2011; Sánchez-Guerra *et al.*, 2012; Gutiérrez-Amavizca *et al.*, 2013; Sandoval-Carrillo *et al.*, 2014; García-González *et al.*, 2015; Jaramillo-Rangel *et al.*, 2015) no reports of the prevalence of these variants in Mexican Amerindian individuals are available. Thus, the aim of this study was to determine and compare the frequencies of *GSTM1* and *GSTT1* null genotypes in Mexican-mestizo and Amerindian individuals (Tarahumara) from the Northwestern part of the country (State of Chihuahua) with those previously found in other regions of Mexico and around the world.

## Materials and Methods

### Study population

The sample population was composed of 422 unrelated individuals from the State of Chihuahua, in the Northwest of Mexico: 211 subjects from the Amerindian ethnic group (Tarahumara) and 211 Mexican-mestizo persons. The Tarahumara sample consisted of 138 females and 73 males with ages ranging from seven to 18 years, whereas the Mexican-mestizo group was composed of 88 females and 123 males, with ages ranging from 16 to 30 years. Samples were collect from July 2009 to March 2014. The Tarahumara group consisted of individuals self-recognized as Amerindians, whose two parents and four grandparents were all born in the locality and speak the Tarahumara language. All participants signed a written informed consent, and in the case of underage individuals, the parents signed their informed consent. Local committees of research ethics approved the study following the Declaration of Helsinki.

### GSTM1 and GSTT1 genotyping

Genomic DNA was isolated from 300 μL of whole blood samples using the MasterPure DNA Purification kit (Epicentre Biotechnologies, Madison, WI, USA), according to the manufacturer’s protocol. DNA integrity was verified by electrophoresis on a 1.2% agarose gel and DNA concentration was evaluated in a Nanodrop 2000 spectrophotometer (Thermo Scientific, Waltham, MA, USA).

Genotyping of null variants in the *GSTM1* and *GSTT1* genes (GenBank accession number: AP000351 and X68676, respectively) was performed by multiplex PCR, as previously described (Arand *et al.*, 1996). Briefly, we used primers to amplify a fragment of the genes *GSTM1* (215 bp), *GSTT1* (480 bp) and the housekeeping *GAPDH* (315 bp), as an internal amplification control, for each sample using a conventional PCR protocol. Also, we used DNA samples with known genotype for *GSTM1* and *GSTT1* null alleles (*GST-T1/M1*: wt/wt, wt/null, null/wt and null/null) as positive controls.

The primers used for PCR amplification were:

*GSTT1*

Forward: 5-TTC CTT ACT GGT CCT CAC ATC TC-3

Reverse: 5-TCA CCG GAT CAT G GC CAG CA-3

*GSTM1*

Forward: 5-GAA CTC CCT GAA AAG CTA AAG C-3

Reverse: 5-GTT GGG CTC AAA TAT ACG GTG G-3

*GAPDH*

Forward: 5-GGA TGA CCT TGC CCA CAG CCT-3

Reverse: 5′-CAT CTC TGC CCC CTC TGC TGA-3′

DNA amplification was carried out with an initial denaturing step at 95 °C for 5 min, followed by 35 cycles of 95 °C for 30 s, 60 °C for 30 s, and 72 °C for 30 s. The PCR reactions were performed in a Veriti 96-well thermal cycler (Applied Biosystems). The PCR products were separated by electrophoresis in 2.5% agarose gels stained with ethidium bromide and visualized by ultraviolet light. In addition, 10% of the samples were genotyped twice from the original DNA sample with a 100% concordance.

### Literature search for genotype data

To identify studies reporting on frequencies of *GSTM1* and *GSTT1* null variants worldwide, a PubMed search was conducted. After excluding meta-analyses and review articles, we considered in our study a total of 57 reports. In order to avoid a bias imposed by the frequency of a gene variant in association with a disease, the frequencies of the null and wild-type genotypes of *GSTM1* and *GSTT1* genes were extracted only from the healthy population reported in each manuscript, but the respective frequencies in the disease-affected population was not considered.

### Statistical analysis

*GSTM1* and *GSTT1* null and wild-type genotypes in Mestizo and Tarahumara populations were reported as frequency. Our findings were compared with those found in other ethnic groups worldwide. The frequencies of the *GSTM1* and *GSTT1* null genotypes were used to generate maps with their geographic distribution using the QGIS 2.4.0-Chugiak shape file (www.naturalearthdata.com). Statistical analysis was performed using the Fisher’s exact test, with *p* < 0.05 considered statistically significant.

## Results

**Figure 1:**
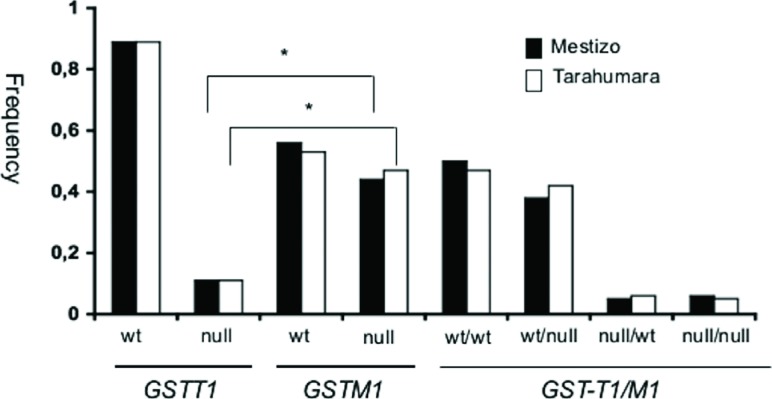
Frequencies of *GSTT1* and *GSTM1* null genotypes in Mestizo (black bars) and Tarahumara individuals (white bars) from the northwestern region of Mexico. Wt: wild type. *p < 0.05.

After genotyping the *GSTM1* and *GSTT1* null polymorphisms, we observed that the *GSTM1* null genotype showed a significantly higher frequency than the *GSTT1* null genotype in both the Mestizo (0.44 *vs.* 0.11) and Tarahumara groups (0.47 *vs.* 0.11). The most common compound genotype in both groups was *GST-T1/M1* wt/wt (Mestizo=0.50; Tarahumara=0.47), followed by the *GST-T1/M1* wt/null genotype (Mestizo=0.38; Tarahumara=0.42). The compound genotypes with lower frequency in both groups were *GST-T1/M1* null/wt (Mestizo=0.05; Tarahumara=0.06) and *GST-T1/M1* null/null (Mestizo=0.06; Tarahumara=0.05) (Figure 1). We found no significant difference in the frequencies of the wild type or of null genotype for *GSTT1* and *GSTM1* between Mestizo and Tarahumara individuals. Likewise, the frequency distribution of the compound genotypes showed no significant difference between Mestizo and Tarahumara individuals.

The frequency of the *GSTT1* null genotype observed in the Mestizo individuals included in our study was similar to those previously reported in Mexican-Mestizos from the northeastern and central regions of the country (0.11 *vs.* 0.10–0.13 and 0.12–0.15, respectively), as well as in one population from the Southeast (0.11 *vs.* 0.09). However, it was significantly higher in comparison with those reported in the western region (0.11 *vs.* 0.03) (Table 1). In the case of *GSTM1*, the frequencies of the null genotype found previously in Mexican-Mestizos from the northeastern and western regions were similar to those of our study (0.44 *vs.* 0.44–0.48, and 0.43, respectively), but populations from the central and southeastern regions showed a significantly lower frequency (0.44 *vs.* 0.33–0.37, and 0.31, respectively) (Table 1). It is worth mentioning that a Mexican-Mestizo population located in the coastal zone of the southeastern region showed the highest frequency of the null genotype for *GSTT1* and the lowest for *GSTM1* in our country (0.17 and 0.22, respectively). These data show a clear reduction in the frequency of the *GSTM1* null genotype from North to South, whereas in the case of the *GSTT1* null genotype no apparent tendency was observed.

**Table 1:** Frequencies of *GSTT1* and *GSTM1* null genotypes in different regions of Mexico.

Region	*n*	*GSTT1*		*GSTM1*		Reference
		wt	null	wt	null	
Northeastern	118	0.87	0.13	0.52	0.48	Jaramillo-Rangel *et al.*, 2015
Northeastern	233	0.90	0.10	0.56	0.44	Sandoval-Carrillo *et al.*, 2014
Northwestern	211	0.89	0.11	0.56	0.44	This study
Western	125	0.97	0.03	0.57	0.43	Gutiérrez-Amavizca *et al.*, 2013
Center	529	0.88	0.12	0.67	0.33	Pérez-Morales *et al.*, 2008
Center	382	0.85	0.15	0.63	0.37	Pérez-Morales *et al.*, 2011
Southeastern	151	0.91	0.09	0.69	0.31	García-González *et al.*, 2015
Southeastern	82	0.83	0.17	0.78	0.22	Sánchez-Guerra *et al.*, 2012

**Figure 2:**
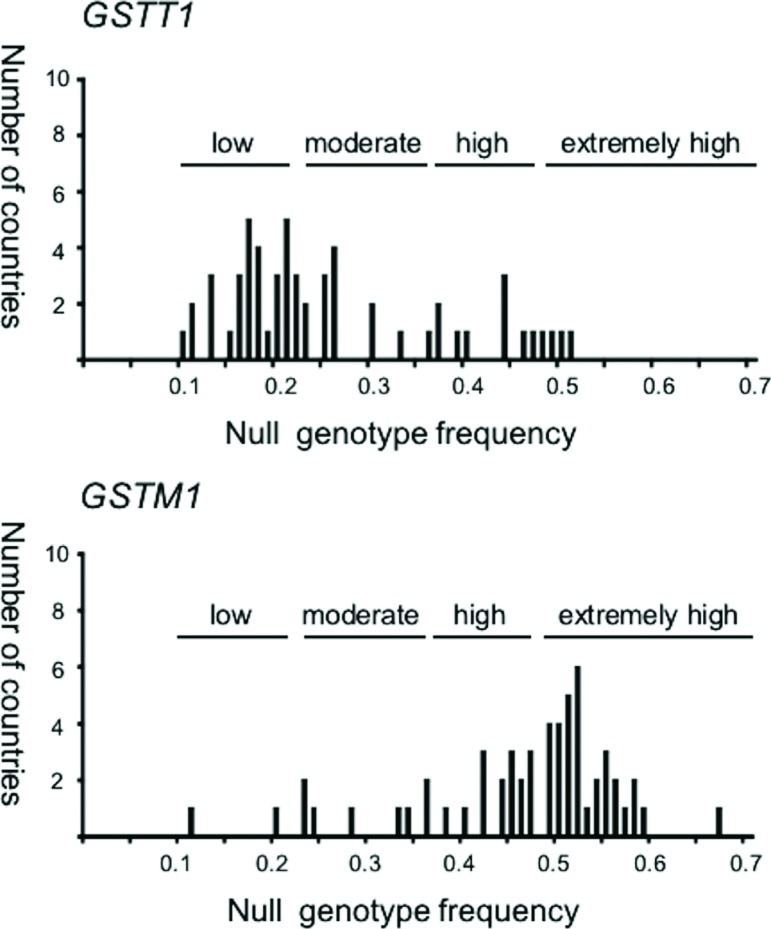
Frequencies of *GSTT1* (upper panel) and *GSTM1* (lower panel) null genotypes in 57 countries.

We also collected from the literature the frequencies of the *GSTT1* and *GSTM1* null genotypes found in 57 countries around the world. The worldwide frequency of the *GSTT1* null genotype ranges from 0.10 to 0.51, whereas that of the *GSTM1* null genotypes ranges from 0.11 to 0.67 (Table 2). To further compare the prevalence of *GSTT1* and *GSTM1* null genotypes worldwide, we classified these frequencies in four groups: common (0.10–0.22), frequent (0.23–0.35), very frequent (0.36–0.48), and extremely frequent (more than 0.48). We observed that the reported frequencies for the *GSTT1* null genotype were common in 31 countries (55%), frequent in 12 (21%), very frequent in 11 (19%) and extremely frequent only in three (5%) (Figure 2, upper panel). In sharp contrast, the reported frequencies of the *GSTM1* null genotype were common in only two countries (3%), frequent in six (10%), very frequent in 17 (30%) and extremely frequent in 32 (56%) (Figure 2, lower panel). Because of the low number of studies reporting frequencies for the compound genotypes, it was not possible make comparisons.

**Table 2:** Frequencies of *GSTT1* and *GSTM1* null genotype in 57 countries worldwide.

Continent/Country		Sample size	*GSTT1*	*GSTM1*	Reference
America	Argentina	69	0.15	0.49	Fundia *et al.*, 2014
	Brazil	137	0.26	0.38	Hatagima *et al.*, 2004
	Canada	274	0.17	0.51	Krajinovic *et al.*, 1999
	Chile	260	0.13	0.42	Acevedo *et al.*, 2014
	Costa Rica	2042	0.20	0.51	Cornelis *et al.*, 2007
	Mexico	211	0.11	0.44	This study
	Paraguay	67	0.18	0.36	Gaspar *et al.*, 2002
	USA	1752	0.21	0.52	Gates *et al.*, 2008
	Venezuela	120	0.11	0.51	Chiurillo *et al*., 2013
	Greenland	100	0.46	0.47	Buchard *et al.*, 2007
Africa	Cameroon	126	0.47	0.28	Piacentini *et al.*, 2011
	Egypt	200	0.30	0.55	Hamdy *et al.*, 2003
	Ethiopia	153	0.37	0.44	Piacentini *et al.*, 2011
	Gambia	337	0.37	0.20	Wild *et al.*, 2000
	Ivory Coast	133	0.33	0.36	Santovito *et al.*, 2010
	Moroco	60	0.22	0.45	Kassogue *et al.*, 2014
	Nambia	134	0.36	0.11	Fujihara *et al.*, 2009
	Saudi Arabia	513	0.25	0.55	Al-Dayel *et al.*, 2008
	Somalia	100	0.44	0.40	Buchard *et al.*, 2007
	South Africa	96	0.20	0.23	Masimirembwa *et al.*, 1998
	Tanzania	220	0.25	0.33	Dandara *et al.*, 2002
	Tunisia	79	0.44	0.46	Ouerhani *et al.*, 2006
	Zimbabwe	150	0.26	0.24	Dandara *et al.*, 2002
Asia	China	763	0.39	0.52	Liu *et al.*, 2009
	India	251	0.16	0.34	Dunna *et al.*, 2013
	Iran	280	0.23	0.49	Rafiee *et al.*, 2010
	Japan	476	0.50	0.52	Hishida *et al.*, 2005
	Korea	1700	0.51	0.54	Kim and Hong, 2012
	Mongolia	207	0.26	0.46	Fujihara *et al.*, 2009
	Philippines	127	0.25	0.59	Baclig *et al.*, 2012
	Singapore	177	0.49	0.67	Chan *et al.*, 2011
	Syria	172	0.17	0.23	Al-Achkar *et al.*, 2014
	Taiwan	574	0.44	0.50	Fujihara *et al.*, 2009
	Thailand	81	0.48	0.58	Klinchid *et al.*, 2009
	Vietnam	100	0.30	0.42	Agusa *et al.*, 2010
Europe	Bulgaria	112	0.16	0.52	Toncheva *et al.*, 2004
	Croatia	60	0.22	0.45	Zuntar *et al.*, 2014
	Czech Rep.	67	0.22	0.57	Binková *et al.*, 2002
	Denmark	537	0.13	0.52	Buchard *et al.*, 2007
	Estonia	202	0.18	0.55	Juronen *et al.*, 2000
	Finland	482	0.13	0.47	Garte *et al.*, 2001
	France	115	0.26	0.49	Abbas *et al.*, 2004
	Germany	3054	0.17	0.52	Kabesch *et al.*, 2004
	Greece	171	0.10	0.52	Dialyna *et al.*, 2003
	Holland	419	0.23	0.50	Garte *et al.*, 2001
	Italy	546	0.17	0.49	Palli *et al.*, 2010
	Lithuania	456	0.16	0.47	Danileviciute *et al.*, 2012
	Poland	365	0.21	0.45	Reszka *et al.*, 2014
	Russia	352	0.19	0.50	Gra *et al.*, 2010
	Serbia	50	0.40	0.56	Stosic *et al.*, 2014
	Slovakia	332	0.18	0.51	Garte *et al.*, 2001
	Slovenia	386	0.21	0.50	Petrovic and Peterlin, 2014
	Spain	461	0.20	0.42	Ruano-Ravina *et al.*, 2014
	Sweden	203	0.18	0.51	Bu *et al.*, 2007
	Turkey	140	0.21	0.55	Aydin-Sayitoglu *et al.*, 2006
	England	1122	0.21	0.58	Garte *et al.*, 2001
Oceania	Australia	1246	0.17	0.54	Spurdle *et al.*, 2007

**Figure 3:**
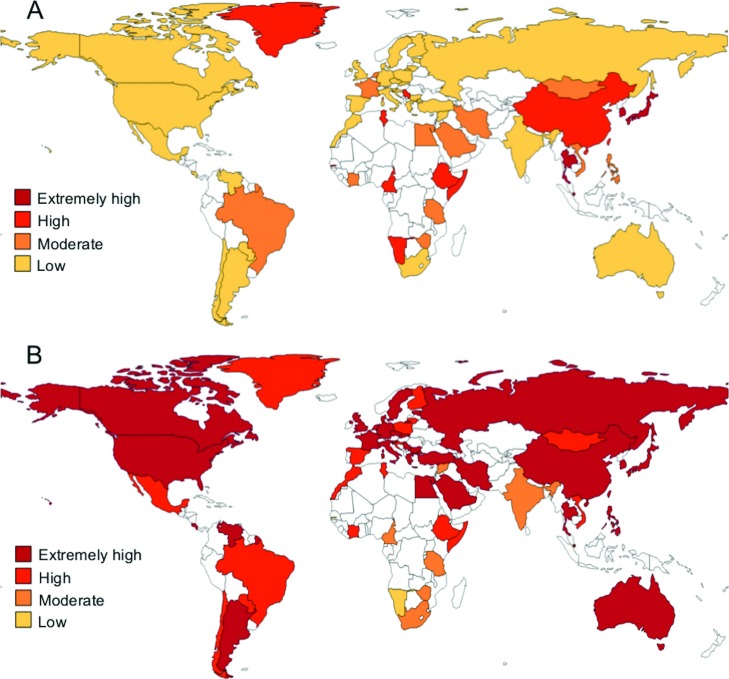
Distribution of *GSTT1* (A) and *GSTM1* (B) null genotypes in 57 countries. Low: 0.10–0.22; moderate: 0.23–0.35; high: 0.36–0.48; extremely high: 0.48–0.67.

Regarding the geographical distribution of these null variants, the countries where *GSTT1* null genotype frequencies were common or frequent were distributed over the five continents, whereas those where the *GSTT1* null genotype was very frequent were concentrated mainly in Africa (Namibia, Gambia, Ethiopia, Tunisia, Somalia and Cameroon) and Asia (China, Taiwan and Thailand). It is worth mentioning that the only three countries with extremely frequent presence of this variant were in East Asia (Singapore, Japan and Korea) (Figure 3A). In the case of the *GSTM1* null genotype, this variant was common in Namibia and Gambia (Africa), and frequent in Syria and India (Asia), and in South Africa, Zimbabwe, Cameroon and Tanzania (Africa), whereas countries with very frequent and extremely frequent frequency were distributed all over the world (Figure 3B).

## Discussion

GST proteins are essential molecules in cellular protection against a myriad of environmental and intracellular compounds. Null variants occurring in the *GSTT1* and *GSTM1* genes are the most common polymorphisms in GST proteins, and their association with various chronic-degenerative diseases such as hypertension, diabetes, asthma, and different types of cancer including prostate, neck, colorectal, liver and leukemia has been thoroughly studied in different populations (Song *et al.*, 2012; Zhang *et al.*, 2012; Liang *et al.*, 2013; Liu *et al.*, 2013; Eslami and Sahebkar*,* 2014; He *et al.*, 2014; Rao *et al.*, 2014; Masood *et al.*, 2015). Both the prevalence of the *GSTT1* and *GSTM1* null genotypes as well as their association with disease phenotypes are highly dependent on ethnic background.

The Mexican-Mestizo population is a complex genetic admixture consisting of Amerindian (56%), Caucasian (41%) and African alleles (3%), with a decreasing Caucasian and an increasing Amerindian ancestry from North to South (Lisker *et al.*, 1986).

In our study, we found no significant difference in the frequencies of the *GSTT1* and *GSTM1* null genotypes among Mexican-Mestizo and Tarahumara individuals from the northwestern region of the country. In addition, we observed a high variability in the frequency of the null genotypes for *GSTT1* and *GSTM1* among the different geographic regions of the country, ranging from 0.03 to 0.17 for *GSTT1* and from 0.22 to 0.48 for *GSMT1* (Pérez-Morales *et al.*, 2008, 2011; Sánchez-Guerra *et al.*, 2012; Gutiérrez-Amavizca *et al.*, 2013; Sandoval-Carrillo *et al.*, 2014; García-González *et al.*, 2015; Jaramillo-Rangel *et al.*, 2015). In the case of *GSTM1*, the frequencies of the null genotypes showed a clear reduction from North to South, whereas the frequency of the *GSTT1* null genotypes showed no apparent tendency. The genetic structure of the Mexican population is very complex and is strongly affected by geographical location. For example, populations located in the northern region near to the US border are characterized by an intense admixture with European-derived populations. In contrast, more than 90% of the Amerindian populations in Mexico (68 ethnic groups) are located in the southern region of the country. As the frequency of the *GSTM1* null genotype is higher in American populations with European ancestry (e.g., USA and Canada) than in Latino American populations (e.g,. Mexico, Chile and Paraguay), it may be possible that the decreasing frequency from North to South of this variant could be caused by the admixture occurring with Caucasian populations.

Regarding the prevalence of the *GSTT1* and *GSTM1* null genotypes worldwide, the *GSTM1* null genotype was very frequent or extremely frequent (0.36 and above) in the majority of the analyzed countries (86%), whereas the *GSTT1* null genotype was common or frequent (from 0.10 to 0.35) in most of the countries (76%). Since the *GSTM1* null genotype is more frequent than *GSTT1* in every country, this indicates that the loss of function of *GSTT1* has a more deleterious effect than *GSTM1*. However, we cannot discard that other GST proteins could replace *GSTM1* but not *GSTT1* function.

The lowest frequencies of the *GSTT1* null genotype were found in America (0.11–0.20), with exception of Greenland (0.46), followed by Europe (0.10–0.26) and Africa (0.20–0.47); the highest frequencies were in Asia (0.16–0.51). For *GSTM1*, the lowest frequencies were observed in Africa (0.11–0.55), followed by Asia (0.23–0.67) and America (0.36–0.52); Europe had the highest frequencies (0.42–0.58). Middle East countries showed lower frequencies for both *GSTT1* and *GSTM1* null genotypes than those from far East Asia (*GSTT1*: 0.16-0.23 *vs.* 0.25-0.51; *GSTM1*: 0.23-0.49 *vs.* 0.42-0.69). Moreover, countries such as Japan, Korea, Singapore and Thailand showed extremely high frequencies for both the *GSTT1* and *GSTM1* null genotypes (Table 2). It is worthy of note that the frequency of the *GSTM1* null genotype found in a Mexican-Mestizo population from the southeastern region, which has a high African ancestry, was very similar to the frequency found in several African populations, including Cameroon, Gambia, and Zimbabwe (0.22 *vs.* 0.28, 0.20 and 0.24, respectively) (Wild *et al.*, 2000; Dandara *et al.*, 2002; Piacentini *et al.*, 2011; Sánchez-Guerra *et al.*, 2012).

In summary, the prevalence of the *GSTT1* and *GSTM1* null genotypes showed a very high diversity, dependent on ethnic background.
